# (In)Equity in Alzheimer’s Clinical Trials: A Long Road Ahead?

**DOI:** 10.1002/alz.090885

**Published:** 2025-01-09

**Authors:** Ana Cláudia de Souza, Wyllians Vendramini Borelli, Pedro Rosa‐Neto

**Affiliations:** ^1^ Hospital Moinhos de Vento, Porto Alegre, Rio Grande do Sul Brazil; ^2^ Universidade Federal do Rio Grande do Sul, Porto Alegre, Rio Grande do Sul Brazil; ^3^ Brain Institute of Rio Grande do Sul (InsCer), PUCRS, Porto Alegre, Rio Grande do Sul Brazil; ^4^ McGill Centre for Studies in Aging/Translational Neuroimaging Laboratory, Montreal, QC Canada

## Abstract

**Background:**

Dementia is a complex condition that affects individuals in various ways around the world, with a particularly high prevalence and incidence in low‐ to middle‐income countries. However, the majority of clinical trials on immunomodulators in Alzheimer’s disease have predominantly been conducted in specific geographic regions and populations. Our objective is to assess the diversity and inclusiveness of the participant samples in terminated phase 2 or 3 clinical trials for anti‐amyloid therapies.

**Method:**

We performed a systematic review for completed and terminated phase 2 or 3 clinical trials for Alzheimer’s disease in ClinicalTrials.gov with results published. We also searched for MEDLINE using “((anti‐amyloid) AND (alzheimer disease[MeSH Terms])) AND (therapy[MeSH Terms] OR (drug) OR (trial))”. We selected only trials using anti‐amyloid therapies regardless of the disease stage (Figure 1. Flowchart). We included all studies that provided sociodemographic characteristics of the sample studied. Variables collected were gender, race, countries where the trial was conducted, and mean years of education.

**Result:**

A total of 16055 individuals over 12 trials were evaluated in this work (ENGAGE, EMERGE, GRADUATE I and II, CLARITY‐AD, A4, EXPEDITION3, ELN115727, CREAD and CREAD 2, TRAILBLAZER‐ALZ 1 and 2). Gender was similarly distributed among studies (8911 females, 55.5%). Participants included were mostly white individuals (13664, 85.1%), with a minority of asian individuals (1259, 7.8%), and black individuals (202, 1.2%). Ethnic origins were demonstrated in 8 studies and only 774 (4.8%) individuals self‐reported as being hispanic. Studies were conducted in 164 countries taken altogether, but included only 25 (15.2%) low‐ and middle‐income countries.

**Conclusion:**

Clinical trials using anti‐amyloid drugs have failed to represent the majority of individuals living with AD‐related cognitive impairment on a global scale. It ultimately increases the disparities presented in the diagnosis and treatment of individuals with dementia. Ideally, anti‐amyloid trials should incorporate the epidemiology of individuals with dementia worldwide. These observations underscore the urgent need for coordinated efforts involving scientists, societies, local governments, and industry to prioritize equity as a central goal in science.

